# Reply to: Scoring the capillary leak syndrome: towards an individualized gradation of the vascular barrier injury

**DOI:** 10.1186/s13613-022-01001-z

**Published:** 2022-03-24

**Authors:** Jakob Wollborn, Ulrich Goebel

**Affiliations:** 1 Department of Anesthesiology, Perioperative and Pain Medicine, Brigham and Women’s Hospital, Harvard Medical School, 75 Francis Street, Boston, MA 02115 USA; 2 Department of Anesthesiology and Critical Care, Medical Center, University of Freiburg, Freiburg im Breisgau, Germany; 3 Faculty of Medicine, University of Freiburg, Freiburg im Breisgau, Germany; 4 Department of Anesthesiology and Critical Care, St. Franziskus-Hospital, Muenster, Germany

We thank Belveyre et al. for their insightful and pertinent comments regarding our study, which aimed at better defining the clinical characteristics of critically ill patients showing capillary leak syndrome (CLS) [1]. As the authors point out correctly, there is no consensus definition for CLS and only limited substantial data breaking down this extremely complex topic [2]. It was our intention to analyze a heterogenous cohort of critically ill patients and to create a scoring system that is easy and feasible in a clinical setting, embracing the pathophysiological mechanism of CLS [3].

Moreover, it was the purpose of this study to evaluate CLS upon arrival in the ICU and on the following days. As a first approach we focused on a heterogenous, postoperative cohort—independent of etiology or previous surgery. We agree that intraoperative factors like excessive blood loss or fluid overload may present a significant impact on the development of CLS in the ICU. However, any correlation between the intra- and postoperative settings were beyond the purpose of our current study. As discussed by the authors, individualized advanced hemodynamic management in the intraoperative period may lead to an altered ICU course, however we doubt that this treatment can be considered a universal standard of care and is utilized anywhere. We agree with Belveyre et al*.* that intraoperative factors like the extent of surgery may be relevant in its potential to trigger CLS. In our study, surgical time was comparable in both groups. Fluid administration and blood loss showed differences: while patients in the No-CLS group had significantly lower blood loss and subsequently needed less fluid administration, CLS patients suffered from a higher blood loss and required more fluid in the following time [3]. Our study was not intended neither powered to identify all potential intraoperative risk factors that may lead to a postoperative CLS, therefore we agree with the authors that the aforementioned factors may clearly be contributory. Identifying risk factors for CLS, especially originating from the intraoperative period, will be an important future goal.

As outlined in the methodology of our manuscript, the ICU physicians judged the presence or absence of CLS using clinical criteria—thus utilizing the only diagnostic, non-invasive and broadly available way to diagnose CLS so far—the clinical view of an experienced ICU physician. This aspect is not only a limitation to our study (and was therefore discussed), but until commonly accepted criteria for CLS exist it will remain problematic in any research on CLS. Therefore, our criteria for patient classification were clearly defined and limited to hemodynamic instability, positive fluid balance, edema formation, and intravascular hypovolemia (i.e., fluid demand). We agree that fluid overload as well as venous congestion among other reasons may falsely lead physicians into diagnosing CLS. However, a positive fluid demand (or intravascular hypovolemia), as e.g., demonstrated by a positive fluid challenge, a passive leg raise or more elaborate diagnostic measures is rarely present in patients with venous congestions. Herein, intravascular hypovolemia presents an important discriminator of patients with volume overload vs. CLS patients. Clinical practice should demonstrate reliable data as an indication and decision basis considering any fluid administration.

Although radiolabeled albumin may provide insight into vascular barrier integrity, we believe that this technique is not feasible beyond specific research questions for very selected patients, if at all. We agree with the Belveyre et al*.* that sublingual intravital microscopy may strengthen the findings in any study evaluating the microvasculature and its characteristics. We have already started to use this technique in our ongoing projects. Regarding the biomarker VE-Cadherin, we intended to evaluate vascular barrier function beyond glycocalyx degradation and endothelial cell stability. However, the predictive properties of VE-Cadherin proved to be less powerful in our cohort using univariate analyses compared to other markers, and therefore it was not included in the subsequent multivariate analysis. In our future studies, we are eager to learn if our findings regarding VE-Cadherin present a cohort-specific phenomenon.

To summarize, in our following and ongoing projects, we are focusing on (a) monitoring our scoring system regarding its predictive value, (b) analyzing CLS in different patient cohorts (e.g., cardiac surgery) with its specific risk factors, and (c) delineating therapeutic approaches for “score-positive” patients in the ICU with respect to their level of capillary leak. We believe, that CLS—while emerging from different circumstances when inflammation and microvascular barrier alterations occur—presents with a common phenotype. The question remains why only a fraction of critically ill patients develops CLS! The philosophers' stone is yet to be discovered.

## Data Availability

Not applicable.

## Abbreviations

CLS: Capillary leak syndrome
ICU: Intensive care unit
